# Biodegradable microneedles for transdermal sitagliptin delivery: in vivo insights into enhanced diabetes management

**DOI:** 10.1007/s13346-025-01991-w

**Published:** 2025-11-03

**Authors:** Rania M. Yehia, Muhammed Ossama, Ghada H. Elosaily, Ahmed M. Fayez, Reham I. Amer, Dalia A. Attia

**Affiliations:** 1 Department of Pharmaceutics and Pharmaceutical technology, Faculty of Pharmacy, The British University in Egypt (BUE), Suez Desert Road, El-Sherouk City, Cairo, 1183 Egypt; 2 Department of Pharmaceutics and Pharmaceutical Technology, Faculty of Pharmacy, Al-Azhar University, Cairo, Egypt; 3 Department of Pharmacology and Toxicology, School of Life and Medical Sciences, University of Hertfordshire hosted by Global Academic Foundation, New Administrative Capital, Cairo, Egypt; 4 Leicester School of Pharmacy, Faculty of Health and Life Sciences, De Montfort University, Leicestershire, UK

**Keywords:** Diabetes, HFFD rats, Hyaluronic acid, Microneedles, Polyvinyl alcohol, Sitagliptin

## Abstract

Microneedles (MNs) offer a promising alternative for transdermal delivery of hydrophilic drugs such as sitagliptin, evading the gastrointestinal side effects associated with oral administration. Thus, the study focuses on the transdermal delivery of sitagliptin, an antidiabetic drug that inhibits dipeptidyl peptidase-4 (DPP-4). The research includes the formulation and in-vitro, ex-vivo, and in-vivo characterization of MNs for sitagliptin delivery. MN molds were fabricated using Stereolithography (3D Printing), and biocompatible MNs were made from hyaluronic acid (HA), chitosan, polyvinylpyrrolidone and polyvinyl alcohol (PVA). Sitagliptin MNs with PVA (SMP) and HA (SMH) exhibited favorable physical and mechanical properties, with drug content of 3.5 ± 0.14 mg/g and 3.8 ± 0.12 mg/g, respectively. SMH showed superior skin penetration due to sharper, stiffer tips. Ex-vivo studies using rat skin indicated that the SMH yielded a higher cumulative permeation (36.58%) compared to SMP (24.04%). Additionally, sitagliptin primarily accumulated in the epidermis, with approximately 56% for SMH and 50% for SMP. In-vivo results demonstrated that both SMP and SMH effectively lowered fasting blood glucose levels, with SMH notably improving postprandial glucose and insulin levels. Overall, HA-based biodegradable microneedles present a promising, minimally invasive strategy for sitagliptin delivery, enabling effective type 2 diabetes management while avoiding its gastrointestinal side effects.

## Highlights

3D printing successfully fabricated microneedle molds for drug delivery.

Biocompatible microneedles (MN) deliver sitagliptin for type 2 diabetes management.

MN showed enhanced skin permeation and drug deposition in ex-vivo studies.

Hyaluronic acid MN improved postprandial glucose and insulin regulation in-vivo.

Minimally invasive MN offer a promising alternative for diabetes management.

## Graphical abstract

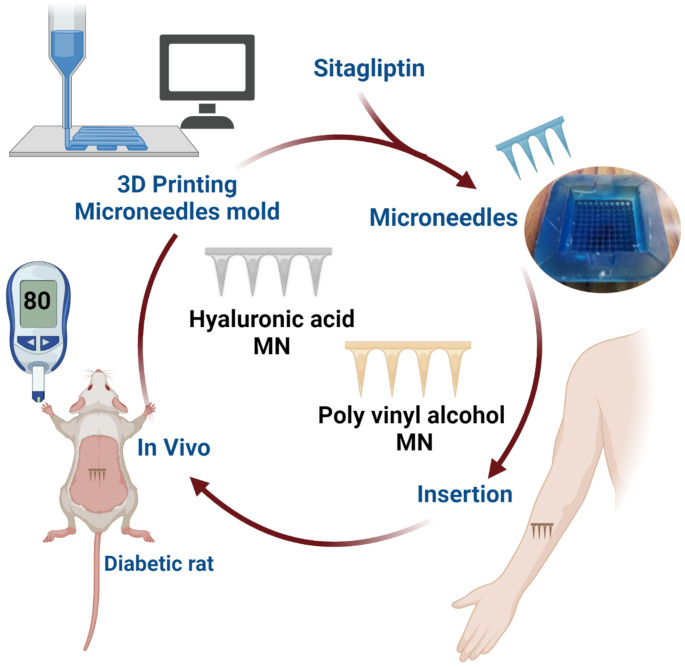

## Introduction

Diabetes is one of the current leading global public health challenges, placing a huge burden on both healthcare systems and socioeconomic development across the world. While the incidence of diabetes in some countries has started to decline, its prevalence in most industrialized and developing nations has continuously risen over the last decades [1]. Diabetes has conventionally been divided into two major types: type 1 and type 2. Type 1 diabetes is of early onset and is characterized by an absolute insulin deficiency resulting from the inability of the pancreas to produce sufficient amounts of insulin. In contrast, the development of type 2 diabetes is more gradual in its onset, and its etiology is directly linked to a condition of resistance to insulin; the pancreas secretes insulin in type 2 diabetes, yet cells do not respond appropriately. Type 2 diabetes can significantly increase the risk for several complications such as cardiovascular disease, kidney damage, visual impairment, neuropathy, and in worst cases, damage to the feet that may lead to amputation [2, 3].

Patients with type 2 diabetes also face the vicious cycle of hyperglycemia and hypoglycemia, where one’s blood glucose levels are either too high or too low. To manage the disease, patients with this illness must continually monitor their glucose levels [4, 5]. The most common modes of treatment for diabetic patients include oral administration and injections of insulin, respectively. Each of these modes of treatment has its drawbacks. Due to the low bioavailability, some drugs are required in high dosage administration in oral medications; this may further cause nausea and abdominal pain as adverse effects. In contrast, insulin injections are to be taken by the patients up to three times a day, which is painful and not very convenient. It also opens up a promising alternative that may relieve diabetes patients from the pain of frequent injections and the adverse effects of oral medications [6–8].

Microneedles (MNs) transdermal patches are made up of a number of microprojections that facilitate the delivery of various drugs and nanoparticle via piercing the top epidermal layer of the skin. The administration of drugs is painless since the microneedles do not reach the nerve fibers [9, 10]. The skin heals within a day or three days post-treatment without the risk of bacterial infection and no prolongation of the discomfort, thus this technique has been employed in delivering vaccines, proteins, small-sized molecules, and cosmetic uses to enhance skin permeability for peptides and proteins [11–13]. Interestingly the market is growing rapidly for the MN technology forecasted to reach $7.8 billion by 2027 [14]. Among all types, polymeric MNs have gained much interest owing to their excellent biocompatibility, biodegradability, and non-toxicity compared to solid MNs prepared from silicon or metals [15]. It is important to note, however, that while most polymeric materials are biocompatible, not all are biodegradable. Synthetic polymers such as PVA and PVP are biocompatible but non-biodegradable, unlike natural polymers such as hyaluronic acid and chitosan, which possess both properties. For safety considerations, low molecular weight grades of PVA and PVP are generally preferred when used in microneedle formulations to allow clearance from the body after application. Also, MNs do not produce harmful sharp waste after use [16]. Polymers with varying degradation profiles and swelling properties enable the fabrication of MNs with varying mechanical properties and functionalities [17, 18].

There are swellable, dissolvable, and biodegradable types of polymers used for MNs. Swellable MNs, like polyvinyl alcohol, expand but do not dissolve, while dissolvable MNs, as the name suggests, made from polysaccharides like dextran, polyvinyl pyrrolidone, sodium alginate or hyaluronic acid, completely dissolve post-application. In contrast to that, biodegradable MNs such as those made from polylactic acid and chitosan, break down in the body over time without swelling or dissolving in the skin [19–21]. Recent studies have investigated microneedles for the delivery of antidiabetic agents such as insulin and metformin, these remain largely within the preclinical or early clinical trial phase, with no microneedle-based systems for diabetes yet reaching the market [7].

Sitagliptin phosphate monohydrate is the first approved DPP-4 inhibitor used as oral hypoglycemic agent in the management of type 2 diabetes, typically taken once daily in a 100 mg dosage [22]. The major hormone implicated in glucose homeostasis is Glucagon-like peptide-1 (GLP-1), responsible for the secretion of insulin from the pancreas. GLP-1 decreases blood glucose by suppressing glucagon secretion, delaying gastric emptying, and may cause satiety. In addition, it is considered to promote beta-cell proliferation and to inhibit beta-cell apoptosis. DDP-4 is an enzyme responsible for the peptidolytic inactivation of GLP-1, and drugs such as sitagliptin inhibit the DDP-4 enzyme, thus increasing active circulating levels of GLP-1. A minimum effective concentration of this drug in the bloodstream has been reported at 100 nM. Sitagliptin, when administered orally, has a number of side effects such as strong stomach pains, respiratory infections, and headaches. These could be avoided, however, in the case of transdermal delivery that reduces gastric overload [6, 22, 23]. Although studies have investigated microneedles for the delivery of antidiabetic agents such as insulin and metformin, these remain largely within the preclinical or early clinical trial phase, with no microneedle-based systems for diabetes yet reaching the market [7].

Thus, the main purpose of this study was to design and evaluate microneedles (MNs) as a novel transdermal delivery platform for sitagliptin, a hydrophilic antidiabetic drug. By employing minimally invasive, dissolvable, and biocompatible polymeric MNs fabricated using stereolithography-based molds. The research seeks to achieve optimal blood concentrations of sitagliptin while avoiding the serious side effects associated with oral administration. The manufactured MNs were assessed through comprehensive in vitro and in vivo methodologies to ensure their efficacy and safety in lowering blood glucose levels.

## Materials and methods

### Materials

Sitagliptin, Chitosan (L.M.W.), polyvinyl alcohol (PVA, M.W. 115000), polyvinyl pyrrolidone k30 (PVP k30), hyaluronic acid (HA), streptozotocin (STZ) and cholesterol were purchased from Sigma-Aldrich (USA). Insulatard^®^ was purchased from Novo-Nordisk (Danish). Glacial acetic acid and glycerol were purchased from Al-Nasr Company for Chemicals and Pharmaceuticals, Cairo, Egypt. Fructose was purchased from UNIPHARMA (Cairo, Egypt), and sheep fat was obtained from a commercial source. All other chemicals used were of analytical grade.

#### Animals

Fifty male adult Wistar rats weighing 120 ± 10 g were obtained from the Animal Production Research Institute, Giza, Egypt. The animals were housed in standard cages under environmental conditions with a temperature of 25 ± 2 °C, which remained constant throughout the experiment. All rats received normal pellet diet and ad libitum water and were allowed to acclimatize for 7 days before initiation of the dietary intervention. The experimental procedure was in accordance with the Helsinki declaration, complying with the ARRIVE guidelines and following the standards of the Guide for the Care and Use of Laboratory Animals. This study also approved the ethical standards set by the research ethics committee of Faculty of Pharmacy-British University in Egypt, number of the approval: EX-2112.

### Methods

#### Mold design and fabrication

**Fig. 1:**
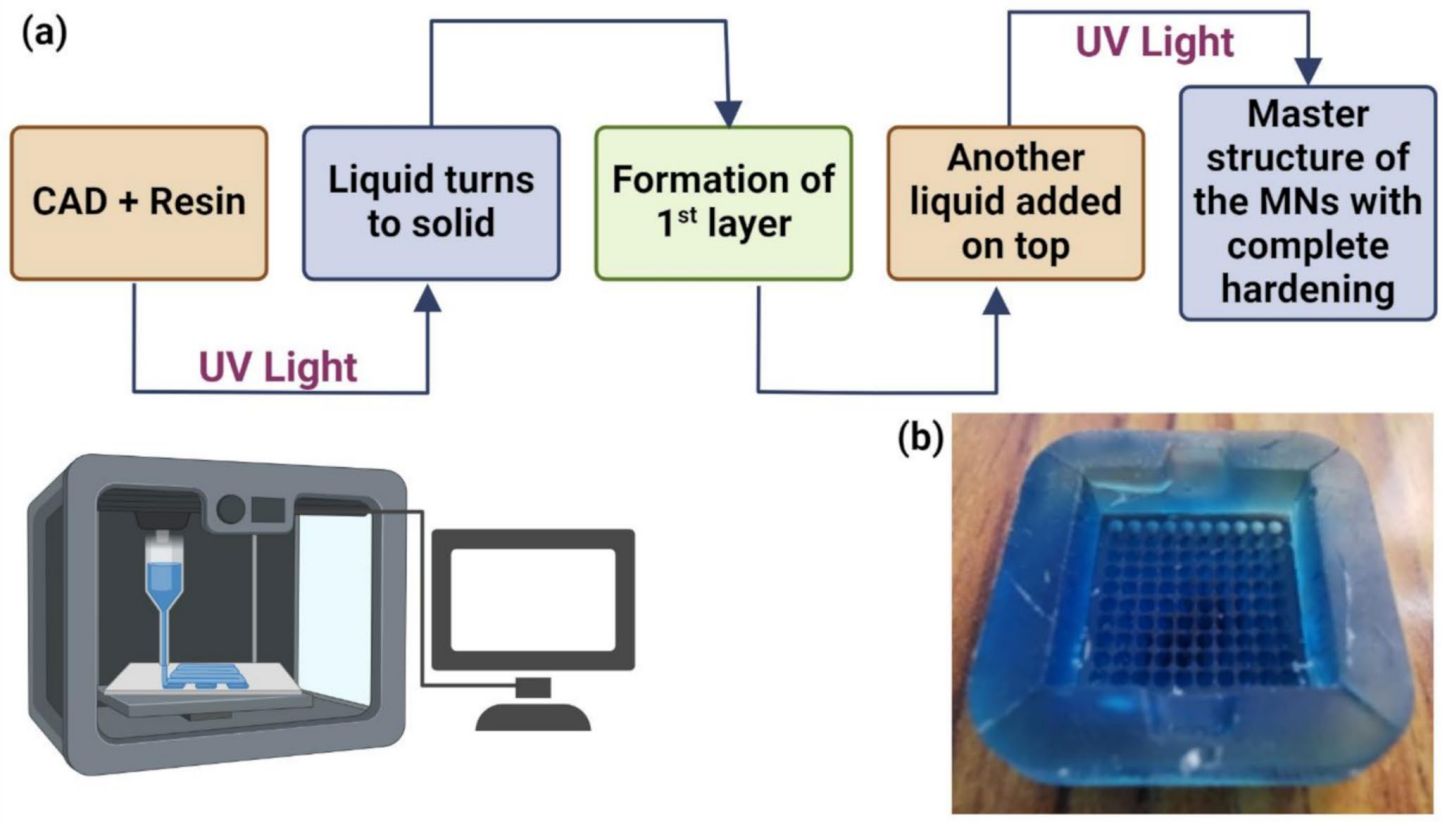
(**a**) Diagrammatic representation of mold fabrication (**b**) Photograph of Stereolithographic (3D printed) fabricated MNs master mold

Computer-aided engineering (CAE) software and Stereolithography (SLA) − 3D Printing were used to design the mold for a 10 × 10 MN array, with an individual needle length of 0.75 mm each, having a base area of 0.045 mm² and an interspacing of 0.01 mm. The two-dimensional digital representation of conical MNs was acquired by using CAE files. The MNs had a conical shape and were fabricated by using MicroChem’s SU-8 photoresist with UV exposure [24, 25]. The process starts when the laser draws the first layer in a photosensitive resin, acting as a stimulus for solidification. After the first layer has been drawn, the platform rises to the value of the thickness of a layer so that more resin can flow under the portion that has just been printed. This laser subsequently solidifies the next cross-section, repeating the process layer by layer until the complete structure is formed. The final product is further cured under UV light to harden it (SLA 3D Printing: Stereolithography 2019, [26]) (Fig. 1a). The master structure of the MNs was then created utilizing various polymers using the constructed mold (Fig. 1b).

#### Fabrication of drug-free MNs arrays

Fabrication of drug-free polymeric MN arrays was conducted using three different types of polymers, at different concentrations, with the addition of varying quantities of polyvinylpyrrolidone (PVP) for each polymer solution (Table 1). Accordingly, each type of polymer was dissolved in the appropriate volume of distilled water, except that chitosan solution was prepared using 1% glacial acetic acid as a solvent. Into the pre-prepared molds, 100 mg of each polymer gel solution was injected using a 1 mL sterile needle-free syringe. Then, the filled molds were further centrifuged at 3500 rpm for 45 min to remove air bubbles and ensure that all pores were completely filled. Then, a second support layer was applied to MN molds and re-centrifuged at 3500 rpm for another 45 min. The support layer, depending on the formulation, comprised a mix of materials: 4% chitosan in a solution of 1% acetic acid for MNC1 to MNC4, 20% PVA for MNP1 to MNP4, and 20% hyaluronic acid for MNH1 to MNH3. All support layers were adapted with the addition of 5% glycerol. MNs arrays were then preserved in dry circumstances following a 24-hour oven drying period at 40 °C. After that, the MNs arrays were carefully removed from the molds [19, 27, 28].

**Table 1:** Formulation of drug-free polymeric MNs arrays

Formulation	Polymers used	Polymer ratio
MNC1	Chitosan: PVP	6:1
MNC2	Chitosan: PVP	4:1
MNC3	Chitosan: PVP	2:1
MNC4	Chitosan: PVP	1:0
MNP1	Polyvinyl alcohol: PVP	6:1
MNP2	Polyvinyl alcohol: PVP	4:1
MNP3	Polyvinyl alcohol: PVP	2:1
MNP4	Polyvinyl alcohol: PVP	1:0
MNH1	Hyaluronic acid: PVP	1:2
MNH2	Hyaluronic acid: PVP	1.5:1
MNH3	Hyaluronic acid: PVP	2.5:1

### Characterization of drug-free polymeric MNs arrays

#### Dimensional analysis

On a disc, a cross-section of simple MNs array was mounted. Using an optical microscope and Image Focus^®^ Software, its morphology was evaluated, and the measurements were taken [29, 30].

#### In-vivo skin penetration

On the surface of human arm skin, the insertion of MNs through the skin was investigated. A fabricated drug-free MNs array was moderately pressed into the skin of a human arm for 10 s using the fingertip. Following application, the MNs array was withdrawn from the skin, and the area was photographed [31]. To stain the hole created in the skin, a methyl-orange dye was applied to the site of array insertion on the skin’s surface for 10 min. The skin pores are then counted.

#### Fabrication of drug loaded MNs arrays

MNH2 and MNP4 were chosen to be loaded with sitagliptin based on the findings of physical evaluation and in-vivo skin penetration testing. In the solutions of the selected polymers, sitagliptin was dissolved. The mold cavities were filled with the drug loaded polymeric solutions. To ensure that the micro-cavities were fully filled, centrifugation was performed at 3500 rpm for 45 min. The same as before, a supporting layer was added. The molds were then dried in an oven at 40 °C for 24 h. Sitagliptin was present in both formulas, sitagliptin MNs HA (SMH) and sitagliptin MNs PVA (SMP) at a concentration of 4 mg/g microneedle. Last but not least, the manufactured MNs arrays included 100 needles that were perpendicular to the conical base [17, 19, 27].

### Characterization of drug loaded polymeric MNs arrays

#### Drug content

The arrays of various formulations were soaked in 25 mL of phosphate-buffered saline (PBS, pH 7.4) until full dissolution in order to ascertain the precise amounts of sitagliptin put into the manufactured MNs. Spectrophotometric analysis was used to quantify the drug content, with absorption maxima occurring at 266 nm [6]. The mean values (*n* = 3 ± SD) were calculated for each sample in triplicate.

#### Ex-vivo drug permeation

 Ex-vivo permeation tests of sitagliptin from polymeric MNs formulations, SMH and SMP, were performed using the Franz diffusion cell, with diffusional area of 3.14 cm^2^. Ventral rat skin was used, and full-thickness skin was placed on the receptor compartment with the stratum corneum facing towards the donor chamber. A cylindrical stainless-steel weight (4 g) was applied for ∼ 5 s to the MN arrays and subsequently removed, thereby allowing the drug loaded MNs to puncture the skin and be positioned in the diffusion chamber. Prior to use, the 20 ml PBS receiver compartment, pH 7.4, was degassed and kept at 37 ± 1 °C. The donor compartment of the diffusion cell was subsequently clamped onto the receiver compartment. Aliquots of 500 µl were removed from the Franz cell at appropriate time intervals and replaced with pre-warmed PBS. Each sample was filtered with 0.45 μm filter paper discs, and the sitagliptin content was assayed. Ex-vivo permeation of sitagliptin from both MNs at 24 h was compared by using one-way ANOVA followed by Tukey’s Multiple Comparison post hoc test; level of significance *P* ≤ 0.05 was considered [32, 33].

#### Mechanical failure force measurement

Sitagliptin from polymeric MNs formulations, SMH and SMP, were subjected to a mechanical failure test utilizing a micro-mechanical test equipment (Instron^®^, model 3345, USA). The axial load that reflects the force applied parallel to the MN array axis was tested to assess the ability of these MNs to bear the force before failure under this load. A moving sensor mount was attached to the MN array, and the mount was moved at 500 mm/min using an axial force. The MNs were forced up against a hard metal surface perpendicular to the mount’s moving axis. The force unexpectedly decreased upon needle failure and the final force applied immediately prior to this decline was noted as the force of MN failure [19, 34]. Data are recorded as the mean values (*n* = 3 ± SD).

#### Skin deposition test

The tape stripping technique was used to explore the levels of sitagliptin deposited in each skin layer. First, the stratum corneum was separated twenty times using adhesive tape to remove the skin. The epidermis and dermis were then separated using a sharp blade. To guarantee full sitagliptin extraction, each skin layer was subjected to six hours of sonication with PBS. A UV spectrophotometer was used for estimating sitagliptin concentrations compared to a blank to avoid any interference [35, 36].

### In-vivo diabetic rat model study

#### Development of type 2 diabetes in rats

This model, adapted from Veerapur et al. (2012), involved randomly dividing 50 male Wistar rats into two different kinds of diet regiments. Six rats were used control, which were fed a standard fat diet containing approximately 3000 cal/kg [3% fats as oil, 21% protein, 60% carbohydrates, 3% fiber, and 3% vitamins] along with normal drinking water. The remaining 44 rats received high-fat and high-fructose diet (HFFD) containing about 5300 cal/kg (3% fats in the form of oil, 15% fats from sheep tail fat, 1% cholesterol powder, 21% protein, 60% carbohydrates, 3% fiber, and 3% vitamins), supplemented with 20% fructose in drinking water. This feeding was carried out for 13 weeks to create insulin resistance. At the start of week 14, the normal fat diet rats were i.p. injected with a single dose of citrate buffer at pH 4.5, while the HFFD group were injected with 35 mg/kg of streptozotocin (STZ) dissolved in freshly prepared citrate buffer at pH 4.5 to induce frank hyperglycemia. To avoid the initial STZ-induced hypoglycemia, 5% glucose in drinking water was given for the first 24 h following the STZ injection. To enhance insulin resistance and decrease the mortality associated with STZ, HFFD rats were treated with long-acting insulin Insulatard^®^ 0.5 IU/kg; i.p. daily for one week. In the beginning of week 15, all rats showing blood glucose above 11.5 mmol/L associated with hypercholesterolemia, hypertriglyceridemia, and hypoinsulinemia one week after STZ treatment were considered T2D rats and selected for further experimentations. Afterwards, all rats received a normal fat diet and fructose-free water until the end of the experiment [37, 38].

#### Parameters assessment

The body weight, the levels of fasting and postprandial serum glucose, triglycerides (TG), total cholesterol (TC), and insulin were recorded weekly during the experiment. Drops of blood have been collected from the tip of the tail vein, test strips for a blood glucose testing device (Bionime^®^) were used to detect blood glucose levels, rat insulin ELIZA kit from MyBioSource (Cat#MBS045315) was used to assess insulin levels, and SPINREACT kit (Girona, Spain) was used to measure TG and TC calorimetrically [39]. GraphPad Prism 8 software (San Diego, CA, USA) was utilized for the statistical analysis, values of *p*-value ≤ 0.05 were considered significant [40].

#### Experimental design

The Six rats who received the normal fat diet served as Group 1: normal group (*n* = 6). Thirty-two HFFD rats were divided into four groups: Group 2: served as type 2 diabetes control (T2D) (*n* = 8); Group 3: treated with sitagliptin oral (SO) (10 mg/kg) (*n* = 8), group 4: treated with SMP (0.5 g MN) (*n* = 8), and group 5: treated with SMH (0.5 g MN) (*n* = 8). The SMH and SMP application was after depilating the dorsal skin hair of the rats in groups 4 and 5, 0.5 g of the microneedles were applied in the center of the dorsal skin of each rat and fixed with a surgical plaster for 24 h. Rats received the treatment for 6 days and were fed a normal diet. After the six days, blood glucose and insulin levels were measured fasting 8 h and 2 h after the meals (postprandial) to evaluate the effects of all the preparations [37].

## Results and discussion

### Fabrication of drug-free MNs arrays

Biocompatible polymeric MNs arrays were chosen and manufactured using a master mold created using the SLA technology in order to provide a minimally invasive system to deliver sitagliptin across the skin and to provide the possibility for continuous distribution. One of the most popular kinds of 3D printing is called SLA, which involves building up a computer-designed shape through the superfine layering of a safe liquid resin. A reservoir containing the liquid resin is used to create individual layers that could be stacked to create the required forms. The polymer selection was based on HA was being biocompatible, biodegradable, and its ability to form sharp microneedles [21]. As for Chitosan it was used for its mucoadhesive and antibacterial properties [41]. While PVP and PVA were chosen for their mechanical strength and solubility profile, enabling rapid drug release [42]. MNH1 and MNH3 formulas were excluded. Due of their poor elasticity, they are exceedingly brittle. Due to the remaining polymeric MNs arrays’ somewhat high Young’s modulus, it was anticipated that they would be mechanically robust [19, 43].

### Characterization of drug-free polymeric MNs arrays

#### Dimensional analysis

**Fig. 2:**
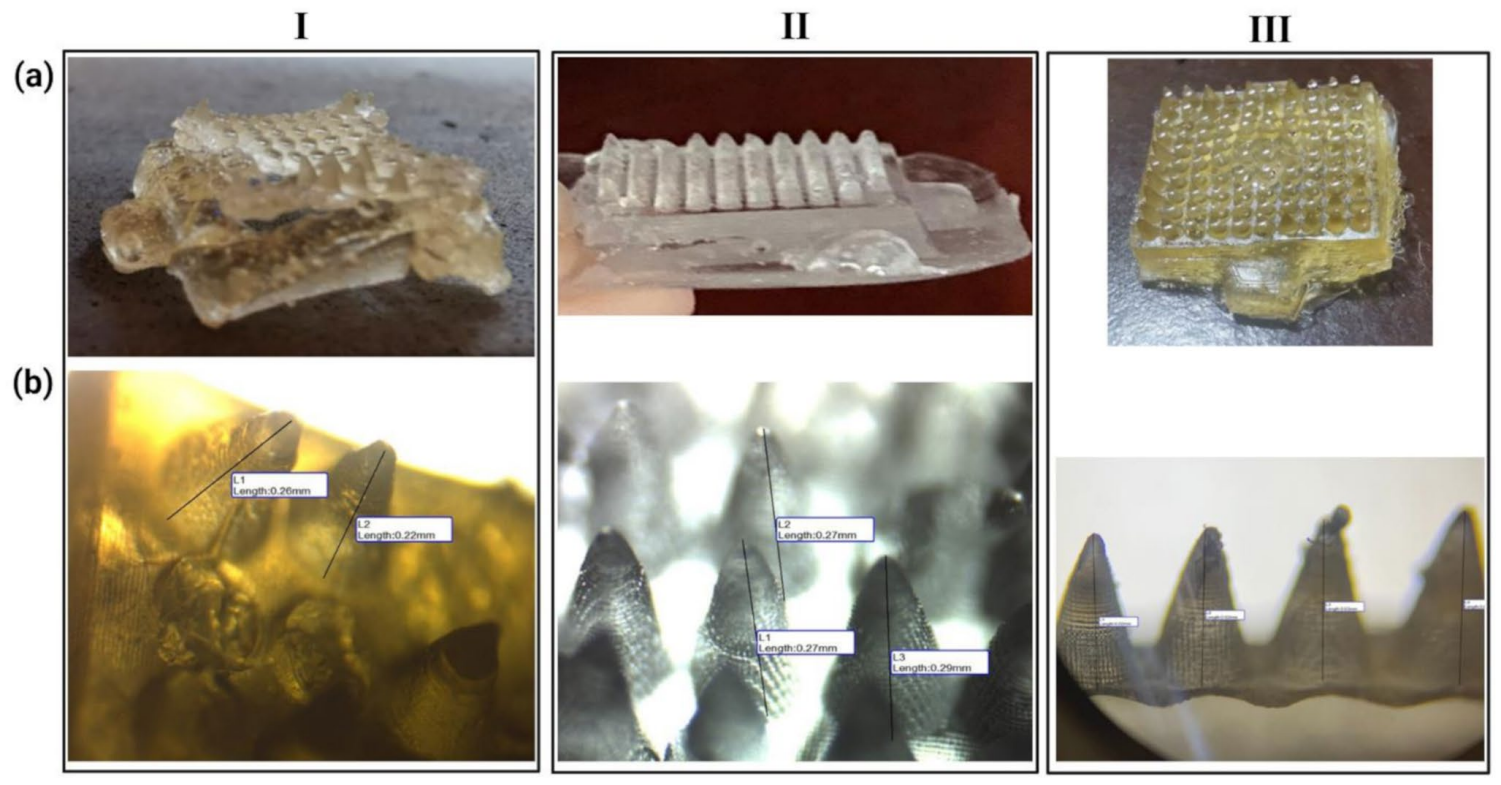
(**a**) Representative photograph of different 10 × 10 polymeric MNs arrays, (**b**) Side view images by Optical microscope with the aid of Image Focus^®^ Software; where (I) Chitosan MN (II) PVA MN and (III) Hyaluronic MN

Figure 2a depicts a generalized view of simple MNs arrays. Using an optical microscope and Image Focus^®^ software, the structural morphology of drug-free MNs was examined (Fig. 2b). The measurements revealed that the basic MNs arrays had a certain geometrical size (Table 2). Although the needles of each individual polymeric MN were near identical in structure to the master mold, the average height of the polymeric MNs alone showed some variation. These may have been due to changes in shape during solidification, possibly dependent on the different viscosities resulting from the various compositions of the polymers. According to [30], that is accurate. Chitosan formulations have the shortest lengths, as seen in Table 2.

**Table 2:** Geometrical dimension of the drug-free MNs arrays and number of stained skin pores formed by insertion of different drug-free MNs arrays

MN formulations	MN length	No. of pores	Tip-base radius (mm)	Area (mm^2^)
MNC1	0.25 mm ± 0.05	3	0.10 ± 0.01	0.03 ± 0.001
MNC2	0.09 mm ± 0.02	0	0.11 ± 0.01	0.04 ± 0.001
MNC3	0.12 mm ± 0.03	1	0.10 ± 0.01	0.04 ± 0.001
MNC4	0.06 mm ± 0.02	0	0.10 ± 0.01	0.03 ± 0.001
MNP1	0.27 mm ± 0.04	12	0.12 ± 0.005	0.05 ± 0.001
MNP2	0.2 mm ± 0.05	4	0.11 ± 0.01	0.04 ± 0.001
MNP3	0.26 mm ± 0.05	11	0.12 ± 0.005	0.04 ± 0.001
MNP4	0.33 mm ± 0.06	74	0.10 ± 0.01	0.03 ± 0.001
MNH2	0.65 mm ± 0.05	88	0.12 ± 0.005	0.04 ± 0.001

#### In-vivo skin penetration

**Fig. 3:**
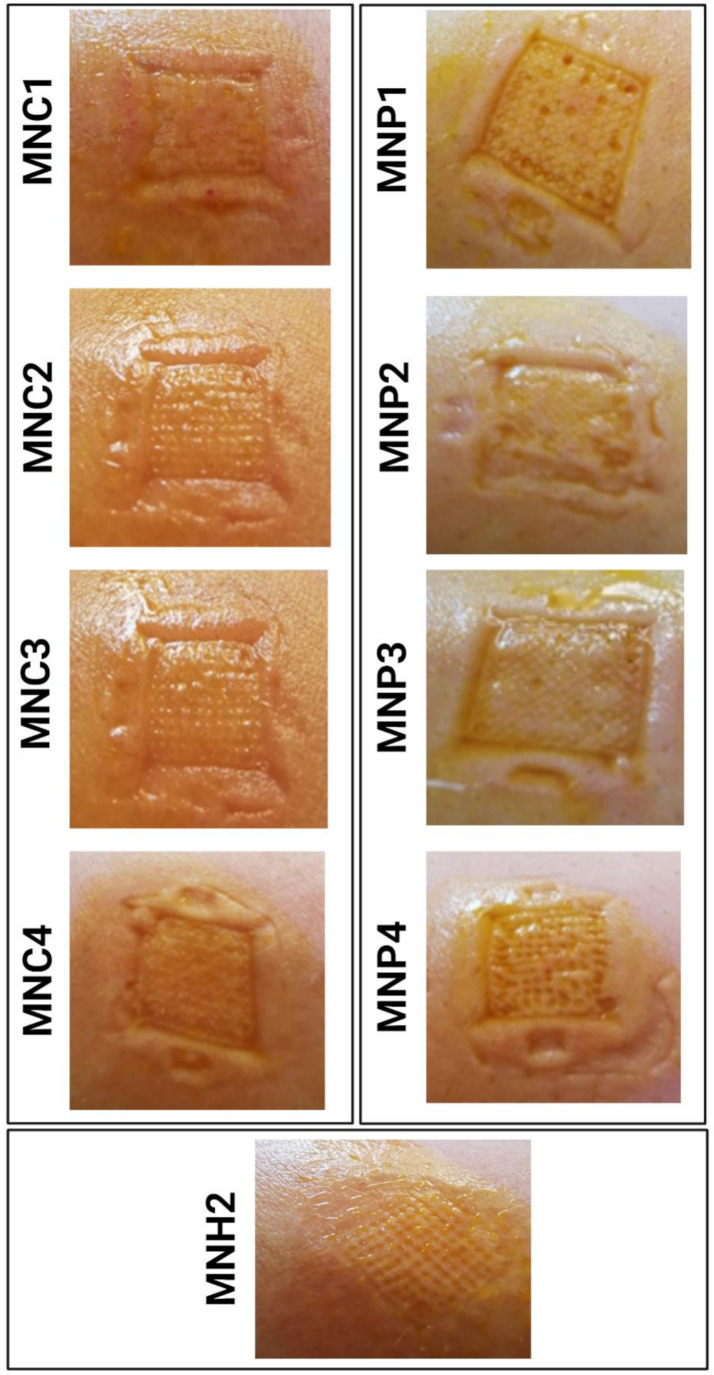
Photograph of stained skin pores formed by insertion of different drug-free MNs arrays; where MNC: Chitosan microneedles (formulae 1 to 4), MNP: PVA microneedles (formulae 1 to 4) and MNH2: Hyaluronic microneedle formula 2

To assess the strength of the MNs, staining and counting of the number of pores formed after the application of MN arrays to human arm skin with constant force was performed. Table 2 shows the number of stained skin pores formed by insertion of MNs arrays. It was noted that in case of chitosan, most likely owing to the low molecular weight chitosan [44]. Besides, the chitosan MNs length was short, which may be one of the reasons that leads to the insufficient penetration, since the effective skin penetration cannot be attained under 0.3-millimeter-long needle length [45]. Regarding to PVA polymer formulations, it is conceivable that by the presence of the PVP, the MNs strength decreased and its ability to penetrate the skin as a result of increased hygroscopic properties and moisture absorption capabilities. On the contrary, PVP hygroscopic nature decreased the time required to dissolve MN arrays and develop cavities in the skin, which absorbed moisture thereby enhancing the dissolution rate of MNs [46]. By comparing the visualized results, Hyaluronic MNs array (MNH2) and PVA MNs array (MNP4) were selected to be loaded with sitagliptin as an optimized drug-free MNs arrays, since both easily penetrated the skin and presented clearly visible micro-pores on the human arm without breakage, as shown in Fig. 3.

#### Drug content measurement

The percentages of sitagliptin in the two formulations of drug loaded MNs were computed. The percentage of sitagliptin in SMP and SMH were 3.5 ± 0.14 mg/gram microneedle and 3.8 ± 0.12 mg/gram microneedle respectively.

#### Mechanical failure force measurement

To ensure that the loaded sitagliptin MNs were sufficiently strong to perforate the skin without breaking, the axial failure force of both SMP and SMH was investigated. Results reported that the axial failure force/MN array of SMH MNs consists of both hyaluronic acid (HA) and PVP in a ratio of (1.5:1) was 7.176 ± 0.6851 N and 4.958 ± 0.4391 N for SMP MNs consist of PVA alone. These displayed results confirm the weaker mechanical strength of SMP compared to SMH MNs, as the polymer–polymer interactions, molecular weight, and formulation ratio may strongly influence mechanical strengthening. Moreover, hyaluronic acid was highly hydrophilic and rich in carboxyl and hydroxyl groups that forms extensive hydrogen bonds with PVP and provides superior hydrogen-bonding networks and matrix compaction, leading to higher needle stiffness and fracture resistance [47]. Additionally, presence of PVP in SMH provides an excellent stabilization, reducing brittleness and improving cohesive strength of the fabricated MNs [48]. These results were in agreement with the number of stained skin pores formed by insertion of different drug-free MNs arrays MNP4 and MNH2 on human arm skin.

#### Ex-vivo drug permeation and skin deposition test

**Fig. 4:**
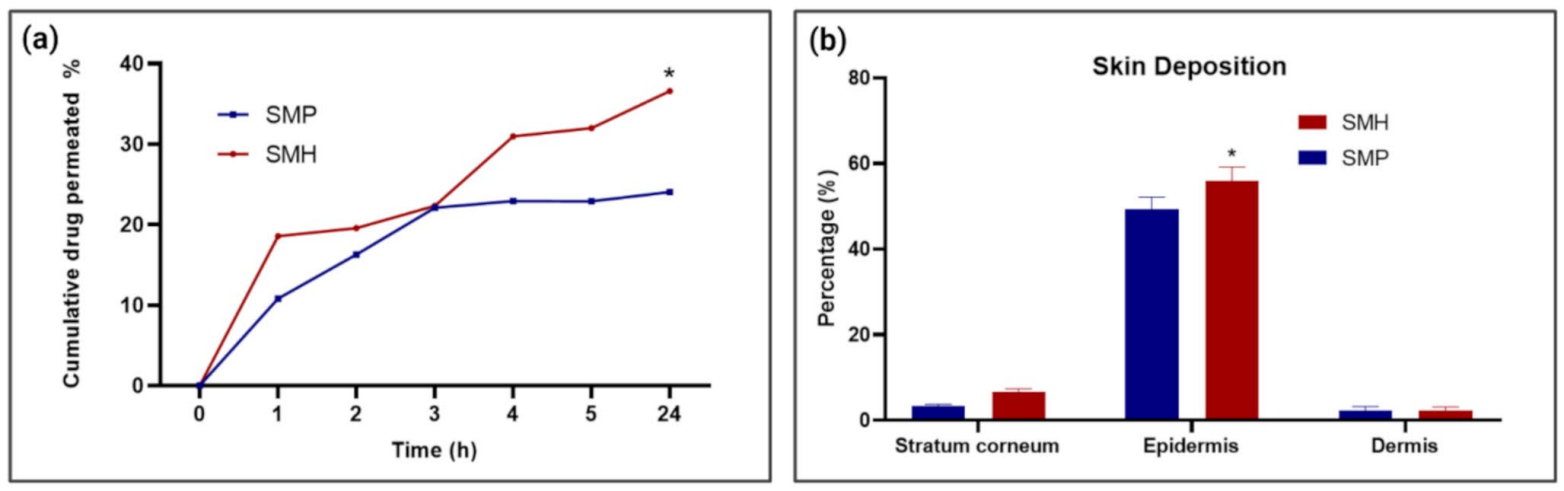
(**a**) Cumulative permeated % of Sitagliptin from drug loaded MNs arrays, (**b**) Amount of sitagliptin deposited in different skin layers, (* indicates a significant value *p* < 0.05)

A study on the two-drug loaded MNs formulae’s ex vivo permeation was conducted. The release medium was PBS buffer (pH 7.4) as it closely resembled extracellular fluids and plasma. Table 3; Fig. 4 demonstrate that, after 24 h, SMP produces a lower cumulative permeated% of sitagliptin (24.04%) than does SMH (36.58%). According to statistical analysis using ANOVA, the permeation of both formulae SMH and SMP after 24 h showed a highly significant difference, *p*-value < 0.001).

**Table 3:** Ex vivo sitagliptin deposition and permeation in different ventral rat skin layers after 24 h

Skin layers	Formulae	
	SMP (% sitagliptin)	SMH (% sitagliptin)
Stratum corneum	3.32 ± 0.43	6.81 ± 0.61
Epidermis	49.40 ± 2.78	56.04 ± 3.2
Dermis	2.36 ± 0.9	2.28 ± 0.87
Cumulative permeated % after 24 h	24.04 ± 0.35	36.58 ± 0.21

**Fig. 5:**
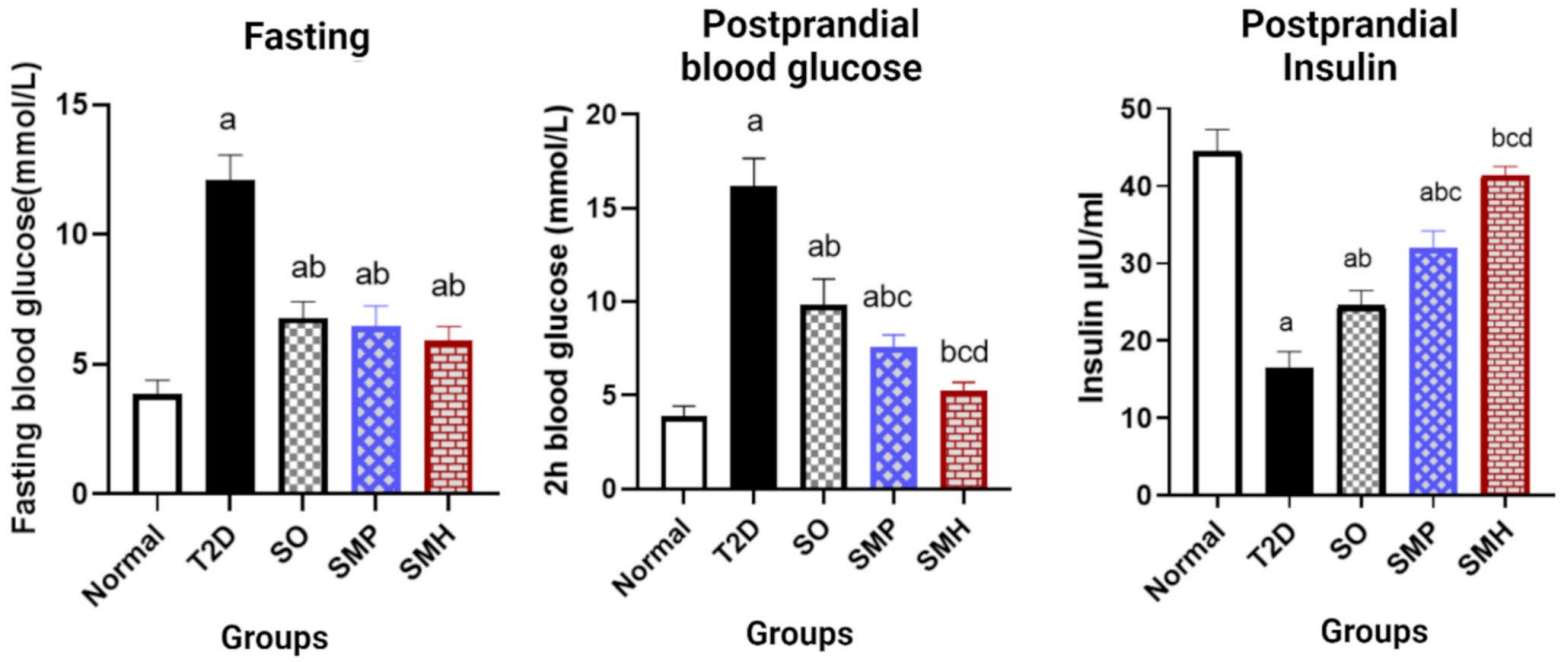
Effect of diabetes and 6 days of administration of sitagliptin oral (SO) (10 mg/kg), sitagliptin microneedle PVA (SMP), and sitagliptin microneedle hyaluronic (SMH) on 8 h fasting blood glucose levels, postprandial blood glucose and postprandial insulin levels. Statistical analysis was performed using one-way ANOVA, followed by Tukey’s post hoc test (*P* < 0.05). Values are expressed as mean ± S.D and a comparison was obtained for a normal group (**a**), T2D (**b**), SO (**c**), and SMP (**d**). T2D: type 2 diabetes; SO: sitagliptin oral; SMP: sitagliptin microneedle PVA; SMH: sitagliptin microneedle hyaluronic acid

The sitagliptin deposition profiles in the stratum corneum, epidermis, and dermis as determined for 24 h after the start of the ex vivo skin deposition investigation carried out using SMP and SMH formulae are shown in Table 3; Fig. 5. Generally, the majority of sitagliptin was found in the epidermis, followed by the stratum corneum and dermis. In comparison to the SMP formula, the sitagliptin amount deposited in the three layers is higher in the SMH formula. The fabricated MNs arrays successfully penetrate the skin’s outermost layer to deliver the medication to the epidermis and the superficial dermis capillary bed.

#### In-vivo diabetic rat model study

Diabetes was induced in the rats of this in vivo study by feeding a high-fat, high-fructose diet (HFFD), that was stopped at the beginning of week 14 when the rats were injected with STZ. The rats were then changed to a normal fat diet (NFD) with fructose-free water for the remainder of the experiment. At the end of the 13th week, the rats fed with HFFD weighed 230 g ± 20, while that of the control group of normal rats were 180 g ± 15.

Type 2 diabetes (T2D) was diagnosed in rats at the beginning of week 15 if their blood glucose levels were more than 11.5 mmol/L and they also had hypercholesterolaemia, hypertriglyceridemia, and hypoinsulinemia. Thirty-two of the forty-four rats in the diabetes group fulfilled these requirements and were chosen for the study. Table 4 shows the statistical analysis performed between normal group and HFFD group after STZ injection using an unpaired student’s t-test, values are expressed as mean ± S.D.

**Table 4:** The analyzed parameters between normal and HFFD rat groups

Groups/Parameters	Normal control	1 week after STZ
Glucose (mmol/L)	4.43 ± 0.51	17.49^a^ ± 1.96
Insulin (µIU/ml)	46.82 ± 2.33	18.68 ^a^ ± 2.24
TG (mmol/L)	3.13 ± 0.31	5.49 ^a^ ± 0.39
TC (mmol/L)	4.28 ± 0.77	8.18 ^a^ ± 1.17

Statistical analysis was performed using an unpaired student’s t−test, where (a) significant difference from the normal control group at (*p* < 0.05). HFFD: high−fat high fructose diet. STZ: streptozotocin

The chosen microneedle (MN) formulations, SMH and SMP (each 0.5 g MN), were then applied to the designated rat groups compared to oral sitagliptin (SO) (10 mg/kg), diabetic and normal controls. Following the topical application of the MNs, the rat skin showed mild erythema after 24 h of microneedle application, but there were no signs of swelling or bleeding, indicating minimal irritation.

After six days of treatment, fasting blood glucose levels (after 8 h fasting) were measured across all groups. As demonstrated in Fig. 5, the treatment groups (SMH, SMP and SO all showed significant difference compared with both the T2D and normal groups. This highlights the efficacy of these treatments in managing fasting glucose levels in diabetic rats.

Next, the postprandial (after 2 h feeding) blood glucose and insulin levels were evaluated, as shown in Fig. 5. The SMH-treated group demonstrated a particularly pronounced effect, with blood glucose levels reduced to near-normal levels. There was no significant difference between the SMH group and the normal group, while the reduction in glucose was significantly greater than in the T2D, SO, and SMP groups. In a similar manner the postprandial insulin levels were high in the SMH group matching those of the normal group and a significant one with the T2D, SO and SMP groups.

The SMP formulation also yielded promising results, with a significant improvement in both blood glucose and insulin levels compared to the T2D and SO groups. However, there remained a statistically significant difference between the SMP group and the normal group, suggesting that while effective, SMP did not fully restore glucose regulation to normal levels.

Herein, these results indicate that the MN formulation, especially SMH, presented better results compared to the oral sitagliptin group, probably because of the direct transdermal delivery of the drug, hence avoiding the first-pass metabolism through the liver. In that way, the drug is more efficiently absorbed and for a longer period of time into the systemic circulation. However, oral sitagliptin is subjected to hepatic metabolism, which may reduce its bioavailability and effectiveness over time, thus accounting for the relatively lower glucose-lowering effect in the SO group as compared to MN-treated groups [49–51].

The SMH formulation outperformed the SMP formulation, such results could be explained from several perspectives. SMH formed of Hyaluronic acid which is a naturally occurring biopolymer with excellent biocompatibility and can improve skin permeability. It allows a deeper and more efficient penetration of drugs in the epidermis the enhance the solubility, swelling index, water vapor permeability [52, 53]. This might be the reason why there was somewhat better drug deposition by the formulation SMH compared to SMP. HA also possesses hydrating and bioadhesive properties, which may enhance residence time of the microneedles on skin, thus allowing for more prolonged drug release and absorption [54]. By comparison, PVA is a common biocompatible polymer, but it has neither the same degree of permeability enhancement nor skin interaction, possibly explaining why SMP had a significant but lesser effect as compared to SMH [53].

Interestingly, no significant differences in the level of fasting blood glucose could be observed within the MN-treated groups. The reason might be that the fasting glucose reflects a basal metabolic rate and endogenous glucose production, which may be less influenced either by short-term intervention or by the capability of the drug to modulate postprandial glucose spikes [55, 56]. Both SMH and SMP had a tendency to reduce fasting glucose levels, though their more impressive effects were related to postprandial glucose regulation, dependent on fast drug absorption and enhancement of the insulin response [57–59]. The superior glucose control of the SMH group further suggests that the enhancement of permeation and facilitation of drug delivery by hyaluronic acid plays a key role in enhancing insulin sensitivity and the metabolism of glucose over postprandial periods.

When placing our findings in the context of other microneedle-based antidiabetic approaches, key differences become apparent. HA-based MNs for insulin delivery in type 1 diabetes have focused on rapid systemic absorption with fast drug release and reversible skin barrier disruption [60]. Similarly, NIR-triggered separable MNs loaded with metformin achieved on-demand release and effective hypoglycemic effects in vivo [61]. In contrast, our sitagliptin-loaded HA microneedles target type 2 diabetes, emphasizing sustained epidermal accumulation and gradual systemic absorption to maintain long-term glycemic control while minimizing gastrointestinal side effects. Other biodegradable MNs, such as alginate–maltose composites delivering insulin, have demonstrated strong hypoglycemic effects close to subcutaneous injection [62], while curcumin-loaded HA MNs for melanoma relied on rapid dissolution for localized therapy [63]. These comparisons underscore the versatility of HA microneedles, which can be tailored to meet distinct pharmacological requirements across different diseases and therapeutic goals.

## Conclusion

This study showed promising results with MNs as an alternative to oral delivery of sitagliptin, which has life-threatening adverse reactions and significantly impairs the quality of life of patients. The MN molds were fabricated by a stereolithography technique, followed by preparation of the MNs loaded with sitagliptin using different biocompatible polymers, such as chitosan, PVP, PVA, and hyaluronic acid. Among these, MNs prepared with PVA and hyaluronic acid had shown the best performances in both in vitro and ex vivo studies by showing excellent mechanical property, drug release, and skin permeation. More importantly, these MN formulations exhibited adequate in vivo efficacy in the management of blood glucose. In particular, the SMH formulation comprising MNs based on hyaluronic acid provided near-normal postprandial glucose and insulin levels in diabetic rats, superior to both PVA-based MNs (SMP) and oral sitagliptin. In conclusion, sitagliptin-loaded MNs developed in the present study represent a promising and safer alternative for the management of glucose homeostasis in type 2 diabetes. This strategy not only enhances the therapeutic efficacy of sitagliptin but also has great potential to distinctly improve the quality of life of millions of patients by reducing adverse effects and offering a minimally invasive, patient-friendly treatment modality. Nevertheless, challenges remain in terms of large-scale fabrication and the need for rigorous clinical validation, particularly to ensure consistent performance and long-term safety in diabetic patients. Further clinical studies are thus merited to fully explore its potential for wide clinical applications.

## Data Availability

The datasets generated or analyzed during the current study are available from the corresponding author upon reasonable request.
